# Modes of e-Health delivery in secondary prevention programmes for patients with coronary artery disease: a systematic review

**DOI:** 10.1186/s12913-019-4106-1

**Published:** 2019-06-10

**Authors:** Gunhild Brørs, Trond Røed Pettersen, Tina B. Hansen, Bengt Fridlund, Linn Benjaminsen Hølvold, Hans Lund, Tone M. Norekvål

**Affiliations:** 1 Department of Heart Disease, St. Olavs University Hospital, Postbox 3250 Torgarden, 7006 Trondheim, Norway; 2 Department of Medicine, Namsos Hospital, Nord-Trøndelag Hospital Trust, Postbox 333, 7601 Levanger, Norway; 3 Department of Heart Disease, Haukeland University Hospital, Postbox 1400, 5021 Bergen, Norway; 4 Cardiovascular Department, Zealand University Hospital, Sygehusvej 10, 4000 Roskilde, Denmark; 5 Department of Regional Health Research, University of Southern Denmark, J.B. Winsløws Vej 19, 3, 5000 Odense C, Denmark; 6 Centre of Interprofessional Collaboration within Emergency Care (CICE), Linnaeus University, 351 95 Växjö, Sweden; 7 Namsos Hospital Nord-Trøndelag Hospital Trust, Postbox, 333 7601 Levanger, Norway; 8 Faculty of Health and Social Sciences, Western Norway University of Applied Sciences, Postbox 7030, 5020 Bergen, Norway; 9 Department of Clinical Science, University of Bergen, Postbox 7804, 5020 Bergen, Norway

**Keywords:** Coronary artery disease, E-health, M-health, Secondary prevention programme, Systematic review

## Abstract

**Background:**

Electronic health (e-Health) interventions are emerging as an effective alternative model for improving secondary prevention of coronary artery disease (CAD). The aim of this study was to describe the effectiveness of different modes of delivery and components in e-Health secondary prevention programmes on adherence to treatment, modifiable CAD risk factors and psychosocial outcomes for patients with CAD.

**Method:**

A systematic review was carried out based on articles found in MEDLINE, CINAHL, and Embase. Studies evaluating secondary prevention e-Health programmes provided through mobile-Health (m-Health), web-based technology or a combination of m-Health and web-based technology were eligible. The main outcomes measured were adherence to treatment, modifiable CAD risk factors and psychosocial outcomes. The quality appraisal of the studies included was conducted using the Joanna Briggs Institute critical appraisal tool for RCT. The results were synthesised narratively.

**Result:**

A total of 4834 titles were identified and 1350 were screened for eligibility. After reviewing 123 articles in full, 24 RCTs including 3654 participants with CAD were included. Eight studies delivered secondary prevention programmes through m-Health, nine through web-based technology, and seven studies used a combination of m-Health and web-based technology. The majority of studies employed two or three secondary prevention components, of which health education was employed in 21 studies. The m-Health programmes reported positive effects on adherence to medication. Most studies evaluating web-based technology programmes alone or in combination with m-Health also utilised traditional CR, and reported improved modifiable CAD risk factors. The quality appraisal showed a moderate methodological quality of the studies.

**Conclusion:**

Evidence exists that supports the use of e-Health interventions for improving secondary prevention of CAD. However, a comparison across studies highlighted a wide variability of components and outcomes within the different modes of delivery. High quality trials are needed to define the most efficient mode of delivery and components capable of addressing a favourable outcome for patients.

**Trial registration:**

Not applicable.

**Electronic supplementary material:**

The online version of this article (10.1186/s12913-019-4106-1) contains supplementary material, which is available to authorized users.

## Background

e-Health is an emerging field of medical informatics, referring to the organisation and delivery of health services and information using the Internet and related technologies [1], such as web-based technology and mobile health (m-Health) [2, 3]. The term e-Health characterises a way of working to improve direct healthcare such as monitoring clinical signs, delivering peer support, as well as health information and education, using information and communication technology [1]. The European Society of Cardiology (ESC) recommends the use of e-Health sources to support remote clinical care and improve psychosocial health, diet and smoking cessation, in both primary and secondary prevention of coronary artery disease (CAD) [2–4]. Secondary prevention programmes, focusing on pharmacological and non-pharmacological treatment, play a pivotal role in decreasing modifiable CAD risk factors as well as improving adherence to treatment and quality of life [4]. Despite the clinical benefits of secondary prevention, only a minority of patients achieve control of modifiable CAD risk factors [5]. It is also known that adherence to treatment in patients with CAD is suboptimal [6]. The reasons for non-adherence are complex and involve factors related to psychosocial factors and social support. Furthermore, secondary prevention programmes are underutilised [7]. Only one-third of patients with CAD attend some form of secondary prevention programme [5]. Geographical accessibility and shortcomings in the healthcare system are main issues for non-participation [7]. This challenge underpins the need for the development of alternative modes of delivery of secondary prevention programmes, such as e-Health programmes where patients receiveaccess to resources at their discretion.

Among the alternative modes of e-Health secondary prevention programme delivery reviewed previously, the individual studies included have primarily focused on telehealth such as telephone interventions [8, 9] and internet technology including web-based and m-Health interventions [10, 11]. The most recent reviews brought some evidence of improved quality of life, diet and physical activity. However, due to the variations between key elements of the studies included, they could not give any implications for practice [10, 11]. A recent updated systematic review on telehealth interventions for patients with CAD, which included more diverse telehealth interventions such as internet and m-Health, reported improved cardiovascular risk factors. However, 60% of the studies included evaluated telephone interventions [12]. To date, telephone interventions are some of the most studied tools of telemedicine interventions [13], but may require more resources than contemporary e-Health technology available. However, the use of e-Health in secondary prevention for CAD is a rapidly growing area and more evidence is therefore expected to become available [11].

Although, a number of components for secondary prevention programmes for CAD have been outlined and standardised in European guidelines and position statements [4, 14, 15], the e-Health secondary prevention programmes seem to differ substantially in their specific components. With regard to the feasibility of e-Health, one systematic review displayed that only 10% of the e-Health secondary prevention programmes included a multicore component approach [16].

To date, no systematic reviews focusing primarily of the effectiveness of m-health and web-based technology as modes of e-Health delivery and its secondary prevention components in patients with CAD have been published. To inform the design of further e-Health secondary prevention programmes, the aim of this systematic review was to describe the effectiveness of different modes of delivery and components for e-Health secondary prevention programmes on adherence to treatment, modifiable CAD risk factors and psychosocial outcomes for patients with CAD.

## Methods

To minimise potential sources of bias this systematic review followed the Preferred Reporting Items for Systematic Reviews and Meta-Analyses (PRISMA) guidelines [17]. The protocol for this systematic review was registered in the International Prospective Register of Systematic Reviews (PROSPERO) [18] (ID: 111927).

### Eligibility criteria

The studies selected had to meet the specific eligibility criteria presented in Table 1. Based on the definition of e-Health [1] and a position statement of the ESC [3], the eligibility criteria regarding the mode of e-Health secondary prevention programme delivery were defined a priori and categorised into: (i) *m-Health* comprising mobile phone applications and/or text messages, but excluding telephone calls (ii) *web-based technology* applicable for traditional desktop or laptop computers; and (iii) a *combination of m-Health and web-based technology*. The modes of e-Health delivery consist of screening, assessment, self-monitoring, self-management, social support, advice and education according to the secondary prevention components.

**Table 1:** Inclusion and exclusion criteria for the studies included in the review

Patient	Adult patients (≥18 years) with CAD
Type of intervention and setting	e-Health secondary prevention programme alone or in addition to traditional secondary prevention care
Comparison	Compared against the group that received traditional secondary prevention care
Mode of e-Health delivery	m-Health; web-based technology; or a combination of m-Health and web-based technology
Secondary prevention components	Physical activity and exercise management; Health education regarding medical; Psychosocial management; Self- monitoring; and Medical risk management
Outcome	Adherence to treatment, modifiable CAD risk factors; psychosocial outcomes
Design	Randomized controlled trials
Exclusion criteria	Interventions in the form of telephone calls; Interventions evaluating heart monitoring systems.

Abbreviations: *CAD* Coronary artery disease, *e-Health* Electronic health, *m-Health* Mobile Health

Based on standardised secondary prevention programme components defined by the British Association of Cardiovascular Prevention and Rehabilitation (BACPR) [19] and a position paper from the European Association for Cardiovascular Prevention and Rehabilitation (EACPR) [14], this review classified a priori the essential secondary prevention programme components into: (i) *Physical activity and exercise management* through exercise plans, supervision and counselling; (ii) *Health education* regarding medical risk management (blood pressure, lipids, glucose) and health behaviour change (dietary habit, weight management, tobacco cessation, psychological management, physical activity); (iii) *Psychosocial management* including counselling on emotional issues, stress management and facilitation of peer support; (iv) *Self- monitoring* of medical risk management (blood pressure, lipids, glucose) and health behaviour change (diet, weight, tobacco cessation, physical activity); and (v) *Medical risk management* including education and support on the use of cardioprotective medications and adherence.

The effectiveness of the eligible studies were described according to three broad categories of a priori defined outcome measures: (i) *Adherence to treatment*, defined as to what extent the patient follows a prescription (e.g. taking medication, following a diet, and/or executing lifestyle changes), that corresponds with agreed recommendations from a healthcare professional [20]; (ii) *Modifiable CAD risk factors* such as high blood pressure, high blood cholesterol levels, smoking, overweight, physical inactivity and poor diet [4, 5]; and (iii) *Psychosocial outcomes*, encompassing depression, anxiety, chronic stress, social support and quality of life [21].

### Search strategy

The search strategy was designed to locate eligible studies published in English over a 15-year period (January 2003 to March 2018). The interval was chosen to reflect the time period in which the most relevant work in e-Health for patients with CAD was published [10]. A team of clinical researchers in the CONCARD^PCI^-study and a PhD-prepared librarian (LBH) agreed upon a search strategy for MEDLINE (Additional file 1: Table S1.), which was then adapted for use in CINAHL and Embase. Articles identified through referenced and hand searches were considered for inclusion.

### Study selection

**Fig. 1:**
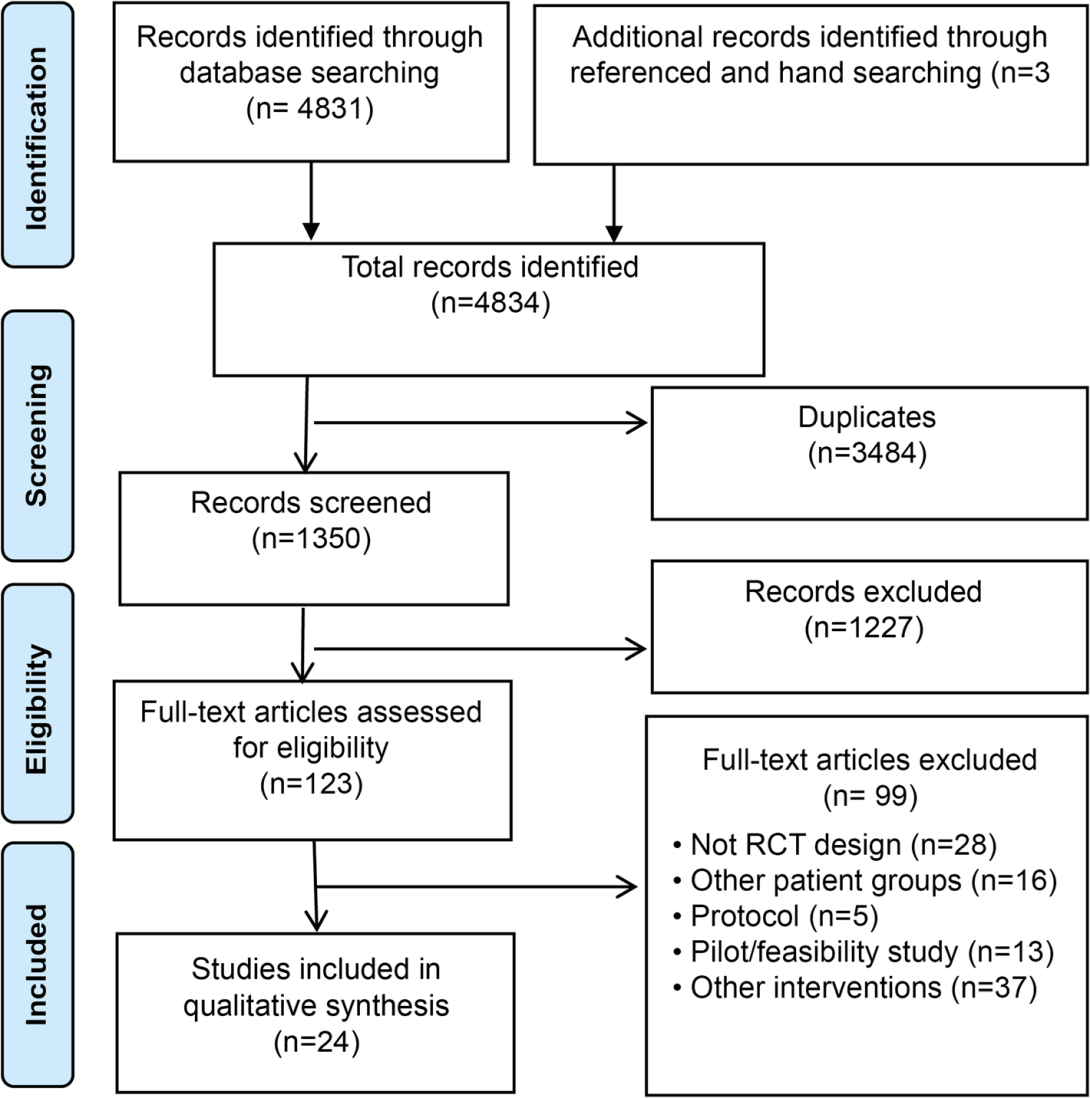
Data selection process and results based on the PRISMA statement [14]

All selected titles and abstracts were scanned independently by two researchers (GB and TRP) using a checklist based on the eligibility criteria (Table 1) to identify papers for potential inclusion. Disagreements regarding the relevance of the abstract were resolved through discussion. The full-text version of the article was obtained and assessed when the abstract information was insufficient, or if one researcher found it necessary to see the full-text version. The full-text versions of the articles were obtained and assessed independently for eligibility by two researchers (GB and TRP). Any disagreements were resolved through discussion or by consulting a third researcher (TMN). The search process shown in the flow diagram was based on the PRISMA statement [17] (Fig. 1).

### Data extraction

The pre-planned data extraction was imported by one researcher (GB) into an Excel spreadsheet. A second researcher (TRP) assessed the data extraction for accuracy. The Cochrane Consumers and Communication Review Group’s data extraction template [22] was used to capture all relevant information about the studies included. Study characteristics were extracted using the Template for Intervention Description and Replication (TIDieR) checklist [23].

### Quality appraisal

The quality appraisal was systematically assessed by two independent researchers (GB and TRP) using the Joanna Briggs Institute (JBI) critical appraisal tool for RCTs. The tool contains 13 items that assess the methodological quality of the design, conduct and analysis of RCTs. The tool comprises four elemental answer choices (Yes, No, Unclear and Not Applicable) [24]. If there was insufficient information to answer a question, unclear was recorded. In non-pharmacological trials, the blinding of participants and health professionals in relation to treatment assessment is not always possible [25]. Therefore, criteria such as the blinding of participants and those who delivered the intervention were considered not applicable (NA) and the performance bias was scored at high risk in all the studies. The results of the quality appraisal assessment are reported in narrative form and in a table for each aspect relating to methodological quality (Table 2).

**Table 2:** Summary of quality appraisal systematically assessed by the Joanna Briggs Institute critical appraisal tool for randomized controlled trials [24]

Author (year)	1	2	3	4	5	6	7	8	9	10	11	12	13	SUM
Norlund et al.2018 [26]	Y	Y	Y	NA	NA	U	U	Y	Y	Y	Y	Y	Y	9
Thakkar et al. 2016 [27]	Y	Y	Y	NA	NA	U	U	Y	U	Y	Y	Y	Y	8
Chow et al.2016 [28]	Y	Y	Y	NA	NA	Y	U	Y	Y	Y	Y	Y	Y	10
Widmer et al. 2017 [29]	Y	U	Y	NA	NA	Y	U	Y	U	Y	Y	Y	Y	9
Johnston et al. 2016 [30]	Y	U	Y	NA	NA	U	U	Y	U	Y	Y	Y	Y	7
Wolf et al. 2016 [31]	U	U	Y	NA	NA	U	U	Y	U	Y	Y	Y	Y	6
Fang et al. 2016 [32]	Y	U	Y	NA	NA	U	U	Y	U	Y	Y	Y	Y	7
Pfaeffli Dale et al. 2015 [33]	Y	Y	Y	NA	NA	N	U	Y	Y	Y	Y	Y	Y	9
Maddison et al. 2015 [34]	Y	U	Y	NA	NA	Y	U	Y	Y	Y	Y	Y	Y	9
Frederix et al. 2015 [35]	Y	U	Y	NA	NA	Y	U	Y	Y	Y	Y	Y	Y	9
Lear et al. 2015 [36]	U	U	Y	NA	NA	U	U	U	U	Y	Y	Y	Y	5
Park et al. 2015 [37]	Y	Y	Y	NA	NA	U	U	Y	Y	Y	Y	Y	Y	9
Park et al. 2014 [38]	Y	Y	Y	NA	NA	U	U	Y	Y	Y	Y	Y	Y	9
Khonsari et al.2015 [39]	U	N	Y	NA	NA	U	U	Y	Y	Y	Y	Y	Y	7
Frederix et al. 2015 [40]	Y	U	Y	NA	NA	U	U	Y	U	Y	Y	Y	Y	7
Devi et al. 2014 [41]	Y	Y	Y	NA	NA	N	U	Y	Y	Y	Y	Y	Y	9
Varnfield et al. 2014 [42]	Y	U	Y	NA	NA	U	U	Y	Y	Y	Y	Y	Y	8
Vernooij et al. 2012 [43]	U	U	Y	NA	NA	U	U	Y	U	Y	Y	V	Y	6
Blasco et al. 2012 [44]	Y	U	Y	NA	NA	U	U	Y	Y	Y	Y	Y	Y	8
Reid et al. 2011 [45]	Y	Y	Y	NA	NA	U	U	Y	U	Y	Y	Y	Y	8
Lindsay et al. 2009 [46]	U	U	U	NA	NA	U	U	U	U	Y	Y	U	Y	3
Vieira et al. 2018 [47]	Y	U	U	NA	NA	U	U	Y	U	Y	Y	Y	Y	6
Vieira et al. 2017 [48]	Y	U	U	NA	NA	U	U	Y	U	Y	Y	Y	Y	6
Southard et al. 2003 [49]	Y	U	Y	NA	NA	U	U	Y	U	U	Y	Y	Y	6

Abbreviation: *NA* Not Applicable, *N* No, *U* Unclear, *Y* Yes

Quality appraisal criteria: [24]

1. Was true randomization used for assignment of participants to treatment groups?

2. Was allocation to treatment groups concealed?

3. Were treatment groups similar at the baseline?

4. Were participants blind to treatment assignment?

5. Were those delivering treatment blind to treatment assignment?

6. Were outcomes assessors blind to treatment assignment?

7. Were treatment groups treated identically other than the intervention of interest?

8. Was follow up complete and if not, were differences between groups in terms of their follow up adequately described and analyzed?

9. Were participants analyzed in the groups to which they were randomized?

10. Were outcomes measured in the same way for treatment groups?

11. Were outcomes measured in a reliable way?

12. Was appropriate statistical analysis used?

13. Was the trial design appropriate, and any deviations from the standard RCT design (individual randomization, parallel groups) accounted for in the conduct and analysis of the trial.

### Describing and analysing studies

Due to large variations between the studies included in terms of key elements (secondary prevention programme components, content delivery, intensity, and outcomes measured between and within the three different modes of e-Health delivery), a meta-analysis was not performed [50]. Further, the studies did not all address multiple risk factors. Using the narrative synthesis methodology, essential modes of delivery and components in e-Health secondary prevention programmes for the effectiveness of outcomes were identified and described. The ESRC methods programme for narrative synthesis was used to collate study findings into a coherent textual narrative, with descriptions of the differences in the studies’ characteristics [51]. The narrative synthesis process was based on the conceptual framework of e-Health secondary prevention programmes, secondary prevention programme components and outcomes defined a priori as described in the section on eligibility criteria. A preliminary synthesis was developed by organising the findings from the studies included to describe patterns across the studies using a textual description. In parallel, the studies were grouped according to the conceptual framework to describe relationships between various modes of e-Health delivery, secondary prevention programme components and the outcome measures of the evaluated studies. Two researchers (GB and TRP) carried out the narrative synthesis process.

## Results

### Characteristics of included studies

The trial selection process identified 4834 records, while 1350 studies were screened for possible inclusion and 123 full manuscripts were reviewed (Fig. 1). In total, 21 completed RCTs in 24 publications that fulfilled the eligibility criteria were included. The studies included were conducted in Asia [32, 39], Europe [26, 30, 31, 35, 40, 41, 43–48], North-America [29, 36–38, 49], and Oceania [27, 28, 33, 34, 42]. A total of 3654 participants (mean age 59.67) were recruited, with the sample size ranging from 46 to 710. Study characteristics are summarised in Table 3.

**Table 3:** Characteristics of studies included in the systematic review of randomized controlled trials assessing the effectiveness of e-Health interventions as part of a secondary prevention programme for patients with coronary artery disease (*N* = 23)

Reference and country	Intervention arm	Control group	Sample size	Population	Mean age (SD)	Men (%)	Outcome measure	Measurement time points	Effects of the intervention	Intervention use
m-health										
Thakkar et al. 2016 [27] Australia.	Text messages in addition to traditional exercise based CR.	Traditional exercise based CR.	710 (IG:352 CG: 358)	Patients with CAD.	57.6 (9.18)	81.9	PO: Physical activity.	Baseline and after 6 months.	Effects on physical activity.	Seven patients requested the text messages to be stopped during follow-up.
Chow et al. 2016 [28] Australia.	Text messages in addition to traditional exercise based CR.	Traditional exercise based CR.	710 (IG:352 CG: 358)	Patients with CAD.	57.6 (9.18)	81.9	PO: LDL-cholesterol level at 6 months. SO: Systolic blood pressure, heart rate, total cholesterol level, BMI, waist circumference, total physical activity, smoking status, proportion achieving guideline levels of modifiable risk factors, and adherence to medications.	Baseline and after 6 months.	Effects on LDL-cholesterol level, systolic blood pressure, BMI and smoking	Seven patients requested the text messages to be stopped during follow-up.
Johnston et al. 2016 [30] Sweden.	An interactive web-based smartphone application and standard secondary prevention care	A simplified Web-based smartphone application and standard secondary prevention care.	174 (IG:91 CG: 83)	Ticagrelor-treated MI patients.	58 (8)	81	PO: Adherence to Ticagrelor, BMI, physical activity, smoking cessation, quality of life. SO: Patient medication use. Quality of life. Tools impact on CV risk factors, use of the tool over time, system usability and satisfaction, safety of the tool.	Evaluated at visit 2, 3 and after 6 months.	Effect on self-reported medication adherence in e-diary.	The proportion of patients who prematurely stopped using the e-diary was low and did not differed between the 2 study groups.
Fang et al. 2016 [32] China.	A: Personalized text messages. B: Personalized text messages and a smartphone application.	Telephone call.	280 (IGa:95;IGb:92 CG: 93)	Patients with chronic stable angina.	53.6	71	PO: Self-reported medication adherence.	Baseline and after 6 months.	No effect	
Park et al. 2015 [37] USA.	A: Text messages for medication reminders and education. B: Text messages for education.	No text messages.	90 (IGa:30 IGb:30 CG: 30)	Patients hospitalized for ACS.	52.9 (9.4)	75	PO: Patient self-reported medication adherence, self-efficacy. SO: Social support, depression.	Baseline and after 30 days.	Effect in the percentage of prescribed number of dose taken, correct doses taken and doses taken on schedule.	
Khonsari et al.2015 [39] Kuala Lumpur.	Text messages medication reminders.	Cardiac rehabilitation and follow-up appointments with cardiologist.	62 (IG:31 CG: 31)	Patients with ACS.	57.9 (12.64)	85.5	PO: The ratio of adherent patients to complete cardiac medication therapy. SO: Heart functional status (NYHA), ACS-related hospital readmission and death rates.	Baseline and after 8 weeks.	Effect in self-reported medication adherence, heart functional status.	93.5% said the system was useful and 64.5% felt that it had helped them taking their medications. 80% requested for the SMS reminders to be continued.
Park et al. 2014 [38] USA.	A: Text messages for medication reminders and education. B: Text messages for education.	No text messages.	90 (IGa:30 IGb:30 CG: 30)	Patients hospitalized for ACS.	52.9 (9.4)	75	PO: Medication adherence.SO: Feasibility and patient satisfaction.	Baseline and after 30 days.	Effect in the percentage of prescribed number of dose taken, correct doses taken and doses taken on schedule.	
Blasco et al. 2012 [44] Spain.	m-health application including telemonitoring and text messages, lifestyle counseling and three clinical visits.	Three clinical visits and lifestyle counseling.	203 (IG:102 CG: 101)	Patients with ACS.	61 (11.5)	83	PO: Cardiovascular risk improvement. SO: Proportion of patients achieving treatment goals, quality of life, anxiety.	Baseline and after 12 months.	Effect in cardiovascular risk factors and treatment goals for blood pressure, BMI, and HbA1c.	Reasons for leaving the programme in the TMG were stress associated with the use of the telemonitoring equipment in 3 patients, personal reasons in 7, and inability to handle the equipment in 2 patients.
Web-based technology										
Norlund et al. 2018 [26] Sweden.	Therapist-guided, tailored Web-based cognitive behavioural therapy. 10 modules with different themes, each containing 2 to 4 treatment steps.	Standard local healthcare.	239 (IG:117 CG: 122)	Patients with a recent MI and symptoms of depression or anxiety.	59.6 (8.49)	67.5	PO: Anxiety and depression. SO: Cardiac anxiety, depression and suicidal ideation.	Baseline and after 14 weeks.	No effect.	Treatment adherence was low.
Vieira et al. 2018 [47] Portugal.	A: Virtual reality programme (Kinect) and education on cardiovascular risk factors. B: Paper booklet and education on cardiovascular risk factors.	Education on cardiovascular risk factors.	46 (IGa:15; IGb:15, CG: 16)	Patients with CAD.	66	100	PO: Executive function. SO: Quality of life, depression, anxiety, stress.	Baseline and after 3 and 6 months.	Effects in executive function for IG1 (selective attention and conflict resolution ability).	The IG1: 82% participated in the first 3 months and 70% in the last three. The IG2: 90% participated in the first 3 months and 75% in the last 3 months.
Vieira et al. 2017. Portugal. [48]	A: A virtual reality programme (Kinect) and education on cardiovascular risk factors. B: A paper booklet and education on cardiovascular risk factors.	Education on cardiovascular risk factors.	46 (IGa:15; IGb:15, CG: 16)	Patients with CAD.	66	100	PO: Bioimpedancce, BMI, waist to hip circumference, and body composition. SO: Physical activity, eating habits, and lipid profile.	Baseline and after 3 and 6 months.	Effects in waist-to-hip ratio, ingestion of total fat and HDL cholesterol level.	The IG1: 82% participated in the first 3 months and 70% in the last three. The IG2: 90% participated in the first 3 months and 75% in the last 3 months.
Lear et al. 2015 [36] Canada.	Virtual CR programme with on-line intake forms, scheduled chat sessions with nurse, exercise specialist and dietitian, education sessions, data capture for stress test and blood test results, monthly ask-an-expert group chat.	Simple guidelines for safe exercising and healthy eating, and a list of internet resources.	78(IG:38 CG: 40)	Patients with CAD.	60	85	PO: Exercise capacity. SO: Lipid profile, blood glucose, Blood pressure, smoking status, BMI, waist circumference, physical activity, diet, hospital admission and emergency room visits.	Baseline and after 4 and 16 months.	Effect in Exercise capacity.	The median number of website logins per person was 27. 122 one-to -one private chat sessions.
Devi et al. 2014 [41] England.	Web-based CR. Tailored goals on exercise, diet, emotions and smoking. Online exercise diary. Feedback on physical activity and smoking. Information on CAD-related risk factors.	Care from the GP and attending an annual check of risk factor management with a nurse.	94 (IG:48 CG: 46)	Patients diagnosed with angina.	66.27 (8.35)	74	PO: Daily average step count, SO: Energy expenditure, duration of sedentary activity, and duration of moderate activity. Weight, blood pressure and body fat percentage, fat and fiber intake, anxiety and depression, self-efficacy, quality of life.	Baseline, 6 weeks after randomization and then 6 months after the 6-week follow-up.	Effect in step-count, energy expenditure, self-efficacy, weight, emotional quality of life score and angina frequency.	The mean number of logins to the program was 18.68, an average of 3 logins per week per participant. Five patients felt trial was too burdensome.
Vernooij et al. 2012 [43] Netherlands.	Internet-based risk factor management programme and usual care.	Physician at the hospital or general practitioner for risk factor management.	330 (IG:164 CG: 166)	Patients with atherosclerosis in the coronary (49%), cerebral or peripheral arteries.	59.9 (8.4)	75	PO: The relative change in Framingham heart risk score after 1 year. SO: The absolute changes in levels of risk factors, differences between groups in the change in proportion of patients reaching treatment goals for each risk factor.	Baseline and after 12 months.	No effect (a relative change of −12% in Framingham heart risk score).	152 patients logged inn at a median of 56 times during the year. Patients (*n* = 134) sent a median 14 messages, and 131 patients entered a median 7 measurements. The monthly number of logins decreased during the intervention period.
Reid et al. 2011 [45] England.	Physical-activity plan and access to a website for planning and tracking, and motivational feedback.	Attending a cardiologist and education booklet.	223 (IG:115 CG: 108)	Patients with ACS.	56.4	84.3	PO: Physical activity: the average number of steps per day. SO: Self-reported leisure-time physical activity, heart disease health-related quality of life.	Baseline, and after 6 and 12 months.	Effects in physical activity, emotional and physical dimensions of quality of life.	61.7% of participants completed at least three of the five tutorials. Thirty-seven participants emailed the exercise specialist at least once.
Lindsay et al. 2009 [46] England.	Moderated web-based discussion groups.	Unmoderated online discussion group.	108 (IG:54 CG: 54)	Patients with CAD.	62.9	66	PO: Changes in health behaviour.	Baseline and after 6 and 9 months.	Effects in self-reported diet during moderated phase.	Message writing to moderators decreased from the moderated to the unmoderated phase, while message writing between participants increased.
Southard et al. 2003 [49] USA	Web-based interactive educational programme	Usual care.	104 (IG: 53 CG: 51)	Patients with CAD.	62.3 (10.6)	75	PO: Diastolic blood pressure, height, weight, LDL levels, exercise, diet, depression, economic evaluation.	Baseline and after 6 months.	Effect on weight loss and BMI,	On average, the individuals in the IG group logged on to the Web site 58 times over the course of the 6-month intervention, or approximately two times per week.
Combination										
Widmer et al. 2017 [29] USA.	Web- and smartphone-based CR in addition to standard phase II CR.	A standard phase II CR.	80 (IG:40 CG: 40)	Patients after PCI for ACS.	62.5 (10.7)	78	PO: CV-related ED visits and readmissions. SO: Weight, blood pressure, heart rate, glucose/HbA1c, lipids, physical activity, diet, quality of life, mood, compliance.	Baseline and after 3 months.	Effect on weight reduction.	16% continued to use the application after 3 months.
Wolf et al. 2016 [31] Sweden	A: Person-centered care in addition to a Web- and mobile-based application. B: Person-centered care.	Usual care.	199 (IGa:37; IGb: 57; CG: 105)	Patients with ACS.	60 (10)	75	PO: Changes in general self-efficacy. SO: Return to work or prior activity level, rehospitalization or death 6 months after discharge.	Baseline and after 6 months.	Effect in general self-efficacy.	The majority used the mobile app rather than the web-based app as the primary source. Patients used the eHealth tool a mean of 38 times during the first 8 weeks and 64 times over a 6-month period.
Pfaeffli Dale et al. 2015 [33] New Zealand.	Personalized text messages and web-page portal in addition to standard CR.	Standard CR.	123 (IG:61 CG: 62)	Patients with CAD.	59.5 (11.1)	81	PO: Adherence to recommended health guidelines, subsequent CAD risk probability. SO: Biomedical risk factors, self-reported medication adherence, self-efficacy, illness perception, anxiety and depression, serious adverse event data.	Baseline and after 3 and 6 months.	Effect on adherence to recommended health guidelines and self-reported medication adherence.	All but one in the IG received the Text4Heart programme. High fidelity to the text messaging component. 85% read all their text messages. 79% felt that 24-week programme was the right length.
Maddison et al. 2015 [34] New Zealand.	Web-site and text messages in addition to community-based CR.	Community-based CR.	171 (IG:85 CG: 86)	Patients diagnosed with CAD.	60 (9.3)	81	PO: Change in PVO2. SO: Self-reported physical activity, self-efficacy and motivation to exercise, health related quality of life. Economic evaluation.	Baseline and after 24 weeks.	Effect in leisure time physical activity and walking, self-efficacy to be active and the general health domain of quality of life.	82% of participants read some or all of the HEART text messages and 57% of participants viewed some or all of the video messages on the web-site. On average participants viewed the website once every 2 weeks.
Frederix et al. 2015 [35] Belgium.	Tele-rehabilitation programme in addition to conventional CR.	Conventional CR.	140 (IG:70 CG: 70)	Patients entered cardiac rehabilitation for CAD or heart failure.	61 (9)	81	PO: VO_2_ peak. SO: Accelerometer-recorded daily step counts, self-assessed physical activity, HbA1c, glycemic control, lipid profile, quality of life.	Baseline and after 6 and 24 weeks.	Effect in VO_2_ peak, self-reported physical activity, and health-related quality of life.	97% patients reported that the telerehabilitation’s motion sensor was easy to read and use. 89% were willing to use the system after study completion.
Frederix et al. 2015 [40] Belgium.	Telemoni-toring and personalized feedback in addition to CR.	CR phase II.	80 (IG:40 CG: 40)	Patients with ACS.	60 (10)	83	PO: Hba1c, lipid profile, VO_2_ peak, waist circumference, blood pressure, BMI. Re-hospitalization.	Baseline, and after 6 and 18 weeks.	Effects in HbA1c, HDL, VO_2_ peak.	
Varnfield et al. 2014 [42] Australia.	Text messages and web-based smartphone application.	Traditional center-based CR.	120 (IG:60 CG: 60)	Post-MI patients referred to CR.	55.7 (10.4)	82	PO: Uptake, adherence and completion of a CR programme. SO: Modifiable lifestyle factors, biomedical risk factors, waist circumference, lipid profile, health related quality of life.	Baseline, 6-weeks and 6-months.	Effects in uptake, adherence and completion rates, quality of life, blood pressure.	

*Abbreviations*: *ACS* Acute coronary syndrome, *BMI* Body mass index, *CAD* Coronary artery disease, *CG* Control group, *CR* Cardiac rehabilitation, *CV* cardiovascular, *HbA1c* Hemoglobin A1c, *HDL* High density lipoprotein, *IG* Intervention group, *LDL* Low density lipoprotein, *MI* Myocardial infarction, *PCI* Percutaneous coronary intervention, *PO* Primary outcome, *PVO2* Peak oxygen uptake, *SO* Secondary outcome, *VO*_*2*_ *peak* Peak aerobic capacity

As regards the theoretical framework, this was described in 10 studies (12 publications); three applied a behavioural change technique [27, 28, 41], two applied the self-efficacy theory of Bandura [37, 38], two applied a patient-centred care approach [29, 31], while the others applied Internet-Based Cognitive Therapy (iCBT) [26], the Chronic Care Model [43], the Social Cognitive Theory [33], and the m-Health Development and Evaluation Framework [34] (Table 4).

**Table 4:** Characteristics of e–Health interventions (*N* = 24) based on the Template for Intervention Description and replication (TIDieR) checklist^22^

Reference	Mode of delivery	Materials	Secondary prevention core components	Theoretical framework	Health professionals	Setting	Duration	Intensity	Effect on outcome i, ii and/or iii
m-health									
Thakkar et al. 2016. Chow et al. 2016 [27, 28]	m-Health.	4 modules with text messages offered information on major secondary prevention areas; physical activity, diet, smoking cessation, general cardiac education.	MM, HE	Behavioural change technique.		e-Health and traditional exercise based CR.	6 months.	96 messages, 1 text message 4 days a week, on random weekdays.	ii
Johnston et al. 2016 [30]	m-Health.	Four main modules: Extended drug adherence e-diary to register daily ticagrelor intake, exercise, weight and smoking. Feedback and information messages General information regarding the cause, symptoms, and treatment of MI.	MM, HE			e-Health and standard secondary prevention care.	6 months.		i
Fang et al. 2016 [32]	m-Health.	Text messages reminders for medications. Micro letter platform which CAD-related education materials (text, images and media content).	MM, HE		A nurse and a physician.	e-Health	6 months.	Educational materials and reminders via the Micro letter platform at regular intervals.	i
Park et al. 2015. Park et al. 2014 [37, 38]	m-Health.	Text messages reminders for medications, text messages education.	MM, HE	Self-efficacy theory.	Nurse.	e-Health	1 month.	Daily text messages reminders, text messages education 3d/week.	i
Khonsari et al.2015 [39]	m-Health.	Text messages reminders for medications, 30-day medication dosage and reminder to come to the hospital and have their prescribed cardiac medication refilled.	MM			e-Health	2 months.	Daily text messages reminders.	i, ii
Blasco et al. 2012 [44]	m-Health.	Biological and clinical data accessed via telemonitoring, individualized short text messages with recommendations including lifestyle counseling.	SM, MM		Cardiologist.	e-Health and and three clinical visits.	12 months.	Patients sent, through mobile phones, biological and clinical data weekly, and subsequently received individualized text messages with recommendations.	ii
Web-based technology									
Norlund et al. 2018 [26]	Web-based technology.	10 treatment modules with 2–4 treatment steps each, homework assignment, feedback, discussion boards, a library with supplementary material and video clips, and text-based psychoeducation. Self-monitoring of mood and daily activities.	HE, PM, SM	Internet-based cognitive behavioral therapy (iCBT).	Psychologist.	e-Health	3.5 months	Patients were recommended to work with one step per week.	
Vieira et al. 2018. Vieira et al. 2017 [47, 48]	Web-based technology.	An exercise protocol, and diary, heart rate monitor, virtual reality exercise programme composed of 3 modules.	PA			e-Health	6 months.	The exercise protocol was performed three times a week over 6 months.	ii
Lear et al. 2015 [36]	Web-based technology**.**	Heart rate monitor and a blood pressure monitor, on-line intake medical, risk factor and lifestyle forms, scheduled one-to-one chat sessions, education sessions (interactive slide presentations), data capture for the exercise test and blood test results, progress notes, and monthly ask-an-expert group chat sessions.	SM, PA, HE		Programme nurse case manager, exercise specialist and dietitian.	e-Health	3 months.	Chat session three times during 12 weeks, weekly education sessions, monthly ask-an-expert group chat sessions, upload their exercise data at least twice per week.	ii
Devi et al. 2014 [41]	Web- based technology.	A online exercise diary recording details of daily exercise, self-monitoring, education on behaviour change techniques, feedback on behaviour goals, information about health consequences, and reducing negative emotions.	HE, SM, PM	Behaviour change techniques.	Cardiac nurses.	e-Health.	1.5 months.	The participants were told to log in daily to record their daily physical activity.	ii, iii
Vernooij et al. 2012 [43]	Web- based technology.	Web page containing risk factor measurements, drug use, treatment goal and advice from the nurse, correspondence between nurse and patient, news items for that particular risk factor. Patients were able to submit new measurements, to read and send messages.	MM, SM, HE	Chronic care model.	Nurse.	e-Health and usual care.	12 months.	The treating nurse practitioner logged in every working day and replied to messages sent by patients and sent messages to patients not using the programme at least every other week.	
Reid et al. 2011 [45]	Web- based technology.	Five tutorials designed to foster behavioral capability, self-efficacy, social support, and realistic outcome expectations. Following each tutorial a new physical activity plan was developed. Between tutorials, participants received emails from the exercise specialist providing motivational feedback.	HE, PM, PA		Exercise specialist.	e-Health.	6 months.	Five online tutorials (at weeks 2, 4, 8, 14, and 20). Each online tutorial took between 10 and 20 min to complete. Asked to log daily activity.	ii, iii
Lindsay et al. 2009 [46]	Web- based technology.	Discussion groups, one-to-one instant messaging with moderators, glossary and information about CAD, diet, exercise and smoking, links and references to local community resources where they could seek help and advice.	PM, HE		Moderator.	e-Health	9 months.	The first 6 months the project were moderated, while the remaining 3 months were unmoderated.	ii
Southard et al. 2003 [49]	Web- based technology.	Web-based program to provide risk factor management support, education, and monitoring services to patients with CVD. Online assessments, online discussion group, a list of participants’ e-mail addresses, and links to related sites on the Internet.	SM, HE		Nurse case managers.	e-Health.	6 months.	Logging on to the site at least once a week for 30 min, communicating with a case manager and dietitian, completing education modules, and entering data into progress graphs.	
Combination									
Widmer et al. 2017 [29]	Combination of m-Health and web-based technology.	Access to health status information, reporting of dietary and exercise habits, graphics showing trends, a social reinforcement networks, educational modules with tasks, targets and plans.	SM, HE, PM	Patient-centered and evidence based material.	Study coordinator.	e-Health and standard phase II CR.	3 months.	Daily tasks, patients were asked to complete educational tasks on a regular basis.	ii
Wolf et al. 2016 [31]	Combination of m-Health and web-based technology.	The mobile app consisted of 3 modules: Self-rated fatigue, symptom trend graph, and built in accelerometer. The web page consisted of 5 modules: Self-rated fatigue, symptom trend graph, diary to capture the everyday experience. Chat function with other patients and nurses, personal links to relevant webpages.	SM, PM	PCC approach.	Nurses and a physician.	e-Health and PCC intervention face-to-face.	At least 2 months.	The patients decided on the frequency and patterns of use of the tool.	iii
Pfaeffli Dale et al. 2015 [33]	Combination of m-Health and web-based technology.	Self-monitoring of physical activity, access to supporting web-page, daily text messages, text an expert to request personalized feedback, health information and recommendation about lifestyle changes via a participant blog, graphs displaying their pedometer step-count, and short video messages from role models and medical professionals.	SM, PM, HE	Social cognitive theory.		e-Health and standard CR.	6 months.	Daily text messages for 13 weeks. From week 13 to 24 the frequency of messages decreased to 5 per week. Self-monitoring of physical activity with pedometer.	i
Maddison et al. 2015 [34]	Combination of m-Health and web-based technology.	Personalized automated package of text messages aimed to increase exercise behaviour, additional information was provided via a web-page including role model video vignettes, an opportunity to self-monitoring progress, and information on various forms of physical activity advice, and links to other website.	PM, SM, HE	The m-Health development and evaluation framework.		e-Health and community-based cardiac rehabilitation.	6 months.	Six text messages per week for the first 12 weeks, five text messages per week for 6 weeks and then four text messages per week for remaining 6 weeks; total 118 messages.	ii, iii
Frederix et al. 2015 [35]	Combination of m-Health and web-based technology.	A patient-specific exercise training protocol, accelerometer for self-monitoring, dietary recommendations, smoking cessation and physical activity tele-coaching strategies, personalized automated feedback emails and text messages encouraging the patients to achieve recommendations.	PA, HE, SM		A health professional who had coached cardiac patients for more than 5 years, supervised by cardiologist.	e-Health and conventional cardiac rehabilitation.	6 months.	Feedback on email and text messages once weekly.	ii, iii
Frederix et al. 2015 [40]	Combination of m-Health and web-based technology.	An accelerometer which registered activity data, personalized automated feedback, emails and text messages designed to encourage the patient to increase daily activity.	PA, HE, SM			e-Health and cardiac rehabilitation phase II.	4.5 months.	Weekly upload of physical activity data. Weekly personalized feedback on physical activity by email or text message.	ii
Varnfield et al. 2014 [42]	Combination of m-Health and web-based technology.	“My heart, My life” manual, health and exercise monitoring, preinstalled audio and video files, motivational and educational materials delivered via text messages.	SM, HE			e-Health	1.5 months.	Weekly scheduled telephone consultations (15 min each), weekly consultations via the web portal to provide informed, personalised feedback on progress according to goals set.	i, iii

Abbreviations: *CAD* Coronary artery disease, *CR* Cardiac rehabilitation, *HE* Health education, *MI* Myocardial infarction, *MM* Medical risk management, *PA/EM* Physical activity and exercise management, *PCC* Person centred care, *PM* psychological management, *SM* Self-monitoring

Outcome: i: Adherence to treatment; ii: Modifiable CAD risk factor; iii: Psychosocial outcomes

Half (54%) of the studies identified in this review described the group of healthcare professionals delivering the e-Health secondary programme, including nurses [37, 38, 41, 43, 49], a nurse and a physician [31, 32], one nurse, exercise specialist and dietitian [36] cardiologist [44], a health professional who had coached cardiac patients for more than five years, supervised by a cardiologist [35], psychologist [26], exercise specialist [45], moderator [46], and a study coordinator [29] (Table 4).

### Description of the e-Health interventions

### m-Health

**Fig. 2:**
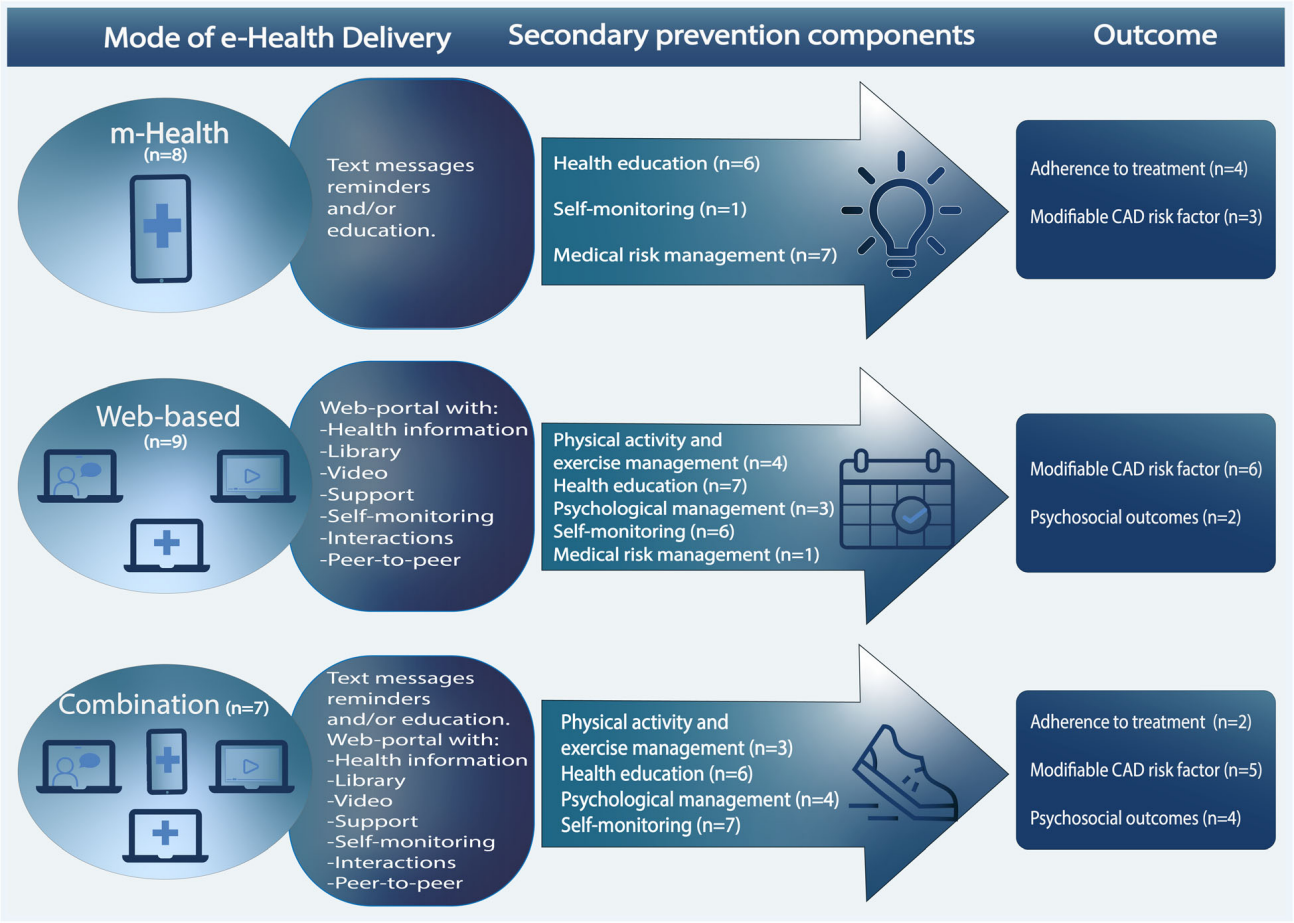
Relationships within and between studies on various modes of delivery, components and significant effect on outcomes of the evaluated studies

Eight studies (*n* = 1513) utilised m-Health technology including text messages, smart phone applications, micro-letter applications and medication reminders to deliver CAD-related health education, recommendations, self-monitoring and medication risk management [27, 28, 30, 32, 37–39, 44] (Tables 3, 4 and 5). Seventy five percent of the studies comprised applications and text messages that provide health education and reminders for medical risk management and/or health behaviour change [27, 28, 30, 32, 37, 38] and one employed medication risk management only [39]. One study only employed self-monitoring [44] (Tables 4 and 5, and Fig. 2). Although text messages were the most common to include, there was a diversity in the materials provided in all the studies. Two studies delivered the m-Health secondary prevention programme in addition to traditional exercise-based cardiac rehabilitation (CR) [27, 28], one in addition to traditional secondary prevention care [30] and clinical visits [44] (Table 4).

**Table 5:** Relationships within and between studies on various modes of delivery and components of the evaluated studies

	Modes of delivery			Secondary prevention components					Outcome measures		
Author (year)	m-Health	Web-based	Combination	PA/EM	HE	PM	SM	MM	Adherence to treatment	Modifiable CAD risk factors	Psychosocial outcomes
Thakkar et al. 2016 [27]	✓				✓			✓		**+**	
Chow et al. 2015 [28]	✓				✓			✓		**+**	
Johnston et al. 2016 [30]	✓				✓			✓	**+**	**–**	–
Park et al. 2015 [37]	✓				✓			✓	**+**		–
Park et al. 2014 [38]	✓				✓			✓	**+**		
Khonsari et al. 2015 [39]	✓							✓	**+**		
Fang et al. 2016 [32]	✓				✓			✓	**–**		
Blasco et al. 2012 [44]	✓						✓			**+**	–
Southard et al. 2003 [49]		✓			✓		✓			**+**	–
Lindsay et al. 2009 [46]		✓			✓	✓				**+**	
Devi et al. 2014 [41]		✓			✓	✓	✓			**+**	+
Norlund et al. 2018 [26]		✓			✓	✓	✓				–
Reid et al. 2012 [45]		✓		✓	✓		✓			**+**	+
Lear et al. 2015 [36]		✓		✓	✓		✓			**+**	
Vieira et al. 2018 [47]		✓		✓							–
Vieira et al. 2017 [48]		✓		✓						**+**	
Vernooij et al. 2012 [43]		✓			✓		✓	✓		**–**	
Widmer et al. 2017 [29]			✓		✓	✓	✓			**+**	–
Wolf et al. 2016 [31]			✓			✓	✓			**–**	+
Pfaeffli Dale et al.2015 [33]			✓		✓	✓	✓		**+**	**–**	–
Maddison et al. 2015 [34]			✓	✓	✓	✓	✓			**+**	+
Frederix et al. 2015 [40]			✓	✓	✓		✓			**+**	
Frederix et al. 2015 [35]			✓	✓	✓		✓			**+**	+
Varnfield et al. 2014 [42]			✓		✓		✓		**+**	**+**	+

Abbreviations: *CAD* Coronary artery disease, *HE* Health education, *MM* Medical risk management, *PA/EM* Physical activity and exercise management; *PM* Psychological management; *SM* Self-monitoring. +: Significant change in outcome; −: No significant change in outcome

Overall, the studies reported secondary prevention programmes delivered by m-Health to be well accepted. In five studies, acceptability was assessed using a feedback questionnaire following completion of the intervention [27, 28, 30, 37, 38]. These studies demonstrated high patient satisfaction with the intervention, where 91% reported it to be useful [28], 88.6% found it easy to use [37, 38], and 97.5% would recommend the intervention to others in the same situation [30]. One study assessed acceptability of the intervention through the number of sessions completed. Adherence to protocol was reported to be high with 83% completing more than 75% of the sessions [44]. Three studies did not provide data on the acceptability of the intervention [27, 32, 39] (Table 3).

### Effect of m-health interventions on outcomes

#### Adherence to treatment

Five studies assessed adherence to medications as the primary outcome [30, 32, 37–39]. There was a non-significant trend towards improved medication adherence in one study [32]. Significant differences were found in four studies [30, 37–39]. One study reported that non-adherence scores were significantly lower in the intervention group compared with the control group when measured with an e-diary (16.6 vs 22.8, *P* = 0.025). However, no difference in self-reported adherence was found at the end of study visit as measured by the Medication Adherence Report Scale (MARS-5) [30]. Regarding antiplatelet medications, two publications reported a higher number of doses taken (*P* = 0.003), correct doses taken (*P* = 0.047) and doses taken on schedule (*P* = 0.004) [37, 38]. Furthermore, one study demonstrated significantly higher medication adherence levels in the intervention group (*P* < 0.001) for patients with acute coronary syndrome as measured by the Morisky Medication Adherence Scale (MMAS-8) [39] (Tables 3, 4 and 5, and Fig. 2).

#### Modifiable CAD risk factors

Four studies utilising m-Health measured modifiable CAD risk factors. There was a diversity of risk factors assessed in the studies. Three studies assessed physical activity [27, 28, 30]. Of these, two studies assessed self-reported physical activity at baseline and follow-up by using the Global Physical Activity Questionnaire (GPAQ) [27, 28]. One study reported higher recreational physical activity (471 vs. 307 metabolic equivalent-min/week, *P* < 0.001), and higher work-related physical activity and lower sedentary times (494 vs. 587 min, P < 0.001) in favour of the intervention group [27]. Further, one study reported that patients in the intervention group exercised more regularly compared with the control group (P < 0.001) [28]. In one study, physical activity was registered in an e-diary. The use of an e-diary did not result in between-group differences in physical activity [30] (Tables 3, 4 and 5, and Fig. 2).

Of the three studies that assessed smoking cessation [28, 30, 44], one study reported significant differences between groups in favour of the intervention group (P < 0.001) [28]. As regards the cardiovascular risk profile, one study reported that more patients in the intervention group achieved treatment goals for blood pressure (62.1% vs 42.9%, *P* = 0.012) and HbA1c (86.4% vs 54.2%, *P* = .0018). In addition, BMI was significantly lower in the intervention group (*P* = 0.005) [44] (Tables 3, 4 and 5, and Fig. 2).

#### Psychosocial outcomes

Three studies utilising m-Health assessed psychosocial outcomes [30, 37, 44]. No significant differences were found for anxiety and depression [44], quality of life [30] or medication self-efficacy [37] (Tables 3, 4 and 5, and Fig. 2).

### Web-based

Nine studies (*n* = 1209) utilised web-based technologies, ranging from web-portals with written information, modules or tutorials regarding CAD-related risk factors, to moderated online discussion groups and chat sessions with health professionals [26, 36, 41, 43, 45–49] (Tables 3 and 4). Seven studies employed health education [26, 36, 41, 43, 45, 46, 49] in combination with physical activity and exercise management [36, 45], psychosocial-management [26, 41, 46], self-monitoring [26, 36, 41, 43, 45, 46, 49] and medication care management [43]. One web-based technology programme employed physical activity and exercise management as a single component [47, 48] (Tables 3, 4 and 5, and Fig. 2). One web-based secondary prevention programme was delivered as an adjunct to usual care [43] (Table 4).

The completion rate of the interventions varied substantially. One study reported that 54% completed the introductory module. Furthermore, 0.9% (1/117) completed the recommended 14 steps within the 14-week treatment period [26]. In four studies, completion rates varied from 43 to 83% [41, 45, 47, 48]. In one study, patient satisfaction was assessed through a semi-structured interview at the end of the programme. In this study, the intervention was perceived to be an accessible, convenient and effective way of delivering healthcare services [36]. Further, one study reported high patient satisfaction, where the patients were ‘very satisfied’ with the programme and reported it to be ‘very helpful’ [49]. One study reported that 152 patients logged in at a median of 56 times during the year. However, the monthly number of logins decreased during the intervention period [43]. Data on acceptability was not reported in one study [46] (Table 3).

### Effect of web-based interventions on outcomes

#### Modifiable CAD risk factors

Seven studies assessed a variety of modifiable CAD risk factors [36, 41, 43, 45, 46, 48, 49]. Six studies assessed physical activity [36, 41, 43, 45, 46, 49]. One study assessed improvement in physical capacity through changes in maximum time on the treadmill stress test (MTT). The intervention group reported a greater increase in MTT by 45.7 s (95% CI: 1.0, 90.5) compared whit usual care (*P* = 0.045) [36]. Two studies [41, 45] assessed physical activity through changes in the average daily step count. In both studies, the intervention group significantly improved their daily step count. One study reported effects from the intervention at the six week follow-up (+ 497 steps), whereas the control group had decreased their number of steps (− 861 steps) (95% CI 263–2451, *P* = 0.02). However, no effects of the intervention were present at six months [41]. Further, one study reported that the intervention group had significantly increased objectively measured (*P* = 0.023) and self-reported physical activity (*P* = 0.047) compared with the intervention group [45]. Two studies utilised self-report alone to assess physical activity. No significant between-group differences were reported [46, 49] (Tables 3, 4 and 5, and Fig. 2).

In terms of the cardiovascular risk profile, systolic and diastolic blood pressure following completion of the programme were reported in two studies [41, 49]. One study reported no significant between-group differences [49], whereas one study reported a significant reduction in systolic blood pressure for the control group compared with the intervention group (ES = 0.68, 95% CI 2.99–13.91, *P* = 0.001) [41]. No significant differences were reported in diastolic blood pressure [41]. Three studies reported cholesterol levels [36, 48, 49]. In one study, intra-group analysis revealed a significant increase in high-density lipoproteins (HDL) in intervention group one (t = − 3.281, *P* = 0.017) [48]. One study reported that total cholesterol and low density lipoprotein (LDL) levels were 7% (*P* = 0.026) and 12% (*P* = 0.022) lower, respectively. However, differences were no longer significant when adjusting for potential confounders [36]. Further, one study found no significant between-group differences in cholesterol levels [49] (Tables 3, 4 and 5, and Fig. 2).

Five studies reported smoking status [36, 41, 45, 46, 49]. Of these, two studies reported smoking status at baseline [36, 41]. One study reported smoking status at baseline and 52 weeks. However, it is unclear whether the results are statistically significant [45]. Two studies found no significant between-group differences [46, 49] (Tables 3, 4 and 5, and Fig. 2).

Two studies assessed weight reduction [41, 49]. Both studies reported significant differences with *P* = 0.003 [49] and P = 0.02 [41]. However, for one study, no effects of the intervention were present at 6-months follow-up [41]. Three studies reported BMI [36, 48, 49]. One study reported a significant reduction in BMI (P = 0.003) [49]. Further, one study found no significant differences in BMI [48], while one study reported the baseline characteristics of participants [36]. Three studies assessed dietary habits [36, 41, 46]. One study reported a decrease in fat intake (ES = 0.30, 95% CI –6.12 to 1.80, *P* = 0.28) and an increase in fibre intake (ES = 0.29, 95% CI–2.23 to 8.53, *P* = 0.25) at six weeks. However, these differences were not present at 6-months follow-up [41]. One study reported that participants in the intervention group had 1.6% kcal/day higher dietary protein and 1.4% kcal/day lower dietary saturated fat, *P* = 0.004 and *P* = 0.018 respectively. These differences remained statistically significant after adjusting for confounders, P = 0.003 and P = 0.018, respectively [36]. One study found no significant between-group differences [46]. One study assessed modifiable CAD risk factors through relative change in Framingham heart risk score at one year. A relative change of − 12% (− 22 to − 3%) in Framingham heart risk score for the intervention group compared with the usual care group was reported. Furthermore, a difference between groups was observed in low density lipoprotein cholesterol (− 0.3, − 0.5 to − 0.1, mmol/L) and smoking (− 7.7, − 14.9 to − 0.4%) [43] (Tables 3, 4 and 5, and Fig. 2).

#### Psychosocial outcomes

Five studies assessed psychosocial outcomes, including anxiety and depression, quality of life and stress [26, 41, 45, 47, 49]. Only two studies demonstrated significant between-group differences in psychosocial outcomes [41, 45]. One study found significant benefits in the social quality of life score (95% CI 0.05–0.54, *P* = .02) at 6-month follow-up in favour of the intervention group [41]. Similarly, another study reported higher emotional dimensions of heart disease health-related quality of life in the intervention group (*P* = 0.038) [45]. One study reported a reduction in anxiety and depression scores over time in the total study sample (mean delta = − 5.1, *P* < .001), but no difference between the study groups at follow-up (beta = − 0.47, 95% CI − 1.95 to 1.00, *P* = 0.53). Furthermore, no effect of treatment was found on the secondary outcomes (severe depression, suicidal ideation, cardiac anxiety) at the follow-up [26]. In two studies, no significant differences were found for quality of life or anxiety and depression [47, 49] (Tables 3, 4 and 5, and Fig. 2).

### Combining m-health and web-based technology

Seven studies (*n* = 913) combined m-Health and web-based technology to deliver health education, self-monitoring and reporting systems, automated feedback from text messages or email, chat functions, and physical activity and exercise management [29, 31, 33–35, 40, 42] (Tables 3 and 4, and Fig. 2). Six studies employed health education [29, 33–35, 40, 42] in combination with: physical activity and exercise management [35, 40], psychosocial-management [29, 31, 33, 34] and self-monitoring [29, 33–35, 40, 42]. One study employed psychosocial management in combination with self-monitoring [31] (Tables 4 and 5, and Fig. 2). Five studies delivered a combination of m-Health and web-based secondary prevention programmes as an adjunct to traditional CR [29, 33–35, 40] and as an adjunct to a person-centred face-to-face care approach [31] (Table 4).

Overall, the acceptability of the combined m-Health and web-based technology programmes was good. One study demonstrated high patient satisfaction, where 95% reported to be ‘satisfied’ or ‘very satisfied’ with the programme [35]. Similarly, two studies reported that 82–85% read some or all text messages [33, 34], and 79% of participants felt that the programme was of the right length [33]. Completion rates of the interventions varied from 73% [29] to 80% [42]. One study reported that 39% chose to use the intervention and continued to use it at least once after discharge from hospital [31]. Data on acceptability was not reported in one study [35] (Table 3).

### Effect of combining m-Health and web-based technology on outcomes

#### Adherence to treatment

Two studies assessed adherence to treatment, more specifically adherence to healthy lifestyle behaviours (primary outcome) and medications (secondary outcome) [33], and adherence to an exercise-based cardiac rehabilitation programme [42]. For the primary outcome, one study reported a significant treatment effect in favour of the intervention at three months (AOR 2.55, 95% CI 1.12–5.84, *P* = 0.3), but not at six months (AOR 1.93, 95% CI 0.83–4.53, *P* = 0.13). For the secondary outcome, medication adherence scores as measured by MMAS-8 were significantly higher in the intervention group (mean difference: 0.58, 95% CI 0.19–0.97, *P* = 0.004) [33]. One study reported that uptake was 1.3 times higher in the intervention group (80%) than in the control group (62%) (*P* < 0.005), the intervention group was 1.4 times more likely to adhere to the programme (RR 1.71; 95% CI 1.13–2.27, P < 0.005), and completion of the programme was 33% higher in the intervention group [42] (Tables 3, 4 and 5, and Fig. 2).

#### Modifiable CAD risk factors

All studies assessed modifiable CAD risk factors. However, there were a diversity of risk factors assessed in the studies. Six studies assessed physical activity [29, 31, 34, 35, 40, 42]. Of these, three studies assessed aerobic capacity (VO_2_ peak) as the primary outcome [34, 35, 40], and one study as the secondary outcome [29]. Two studies reported a significant increase in VO_2_ peak in favour of the intervention group, *P* < 0.001 and *P* = 0.013, respectively [35, 40] whereas two studies reported no significant between-group differences (difference − 0.2 ml^− 1^kg min ^− 1^, 95% CI: -1.1, 0.7: *P* = 0.65) [34] and *P* = 0.67 [29]. However, significant improvements in self-reported leisure time physical activity (difference 110.2 min/week, 95% CI: _0.8, 221.3; *P* = 0.05) and walking (difference 151.4 min/ week, 95% CI: 27.6, 275.2; *P* = 0.02) at 24 weeks were reported in one study [34]. One study used the six-minute walk test (6MWT) to assess physical capacity. Both intervention groups showed significant improvements in 6MWT from baseline to 6 weeks (TCR: 537 ± 86–584 ± 99 m; CAP-CR: 510 ± 77–570 ± 80 m), which was maintained at 6 months. However, between-group differences for changes in 6MWT were not significant at 6-month follow-up [42]. One study reported no significant between-group differences regarding physical activity [31] (Tables 3, 4 and 5, and Fig. 2).

Three studies assessed weight reduction [29, 35, 40]. One study reported a significant difference in weight in favour of the intervention group (− 5.1 ± 6.5 kg vs. − 0.8 ± 3.8 kg, P = 0.02), which was mirrored in the results for BMI and waist circumference [29]. Two studies reported no between-group differences regarding weight, BMI or waist circumference [35, 40] (Tables 3, 4 and 5, and Fig. 2).

Five studies reported systolic and diastolic blood pressure at the end of the programme [29, 33, 35, 40, 42]. One study reported significant improvements in diastolic blood pressure (*P* = 0.03), but not in systolic blood pressure (*P* = 0.4) [42]. Four studies reported no significant between-group differences in systolic or diastolic blood pressure [29, 33, 35, 40].

Five studies reported HDL and LDL cholesterol levels [29, 33, 35, 40, 42]. However, no studies reported significant between-group differences for these parameters. Similarly, no significant between-group differences were reported regarding HbA1c [29, 35, 40].

One study reported a significant treatment effect in favour of the intervention group regarding adherence to recommended health guidelines at three months (AOR 2.55, 95% CI 1.12–5.84; P = 0.03), but not at six months (AOR 1.93, 95% CI 0.83–4.53; *P* = 0.13) [33] (Tables 3, 4 and 5, and Fig. 2).

#### Psychosocial outcomes

Six studies assessed psychosocial outcomes, including anxiety and depression, self-efficacy, quality of life and stress [29, 31, 33–35, 42]. One study reported significant improvements in global health-related quality of life for the intervention group (P < 0.001). Between-group comparison confirmed that the intervention group improved more than the control group (U = 2407, z = 2.805, *P* = 0.01) [35]. One study reported reductions in psychological distress as measured by Kessler 10 (K10) Psychological Distress Scale (median (IQR) 14.6 (13.4–16.0) to 12.6 (11.5–13.8)), as well as significant improvements in the EQ5D-Index for the intervention group at the end of the programme (six weeks). However, these differences were not significant at 6-month follow-up [42]. One study reported a significant improvement in mean self-efficacy levels as measured by the General Self-Efficacy Scale (GSES) for the intervention group (*P* = 0.011) [31]. Further, one study reported significantly higher quality of life scores as measured by the Dartmouth Quality of Life Index (P = 0.03). However, no significant differences were found regarding stress and depression [29]. One study reported significant improvements in self-efficacy to be active (difference 6.2, 95% CI: 0.2,12.2; *p* = 0.04) and the general health domain of the SF-36 (difference 2.1, 95% CI: 0.1, 4.1; *p* = 0.03) [34]. Furthermore, one study reported a negative effect for total hospital anxiety as the intervention group reported greater anxiety than the control group at six months (mean difference: 1.18, 95% CI 0.28–2.08, P = 0.01) [33] (Tables 3, 4 and 5, and Fig. 2).

### Quality appraisal

On average, the studies included had a moderate methodological quality, with scores ranging from three to 13 (median 8) (Table 2). True randomisation was used for the assignment of participants to treatment groups in 21 studies. In three studies, the randomisation procedure was insufficiently reported (selection bias) [31, 39, 46]. The major risk of bias in the studies included was due to insufficuent blinding of participants, those who delivered the intervention (performance bias) and assessors (measurement bias). There was also a risk of allocation bias due to the treatment of interest. Half of the studies included in the review reported that participants were analysed in the groups to which they were randomised (attribution bias). The detailed quality appraisal of the studies included, systematically assessed by use of the JBI critical appraisal tool for RCTs [24], is given in Table 2.

## Discussion

The current systematic review identified, appraised and synthesised 24 publications that evaluated e-Health secondary prevention programmes and their effects on adherence to treatment, modifiable CAD risk factors and psychosocial outcomes for patients with CAD. The quality of these studies was moderate (median score of eight as measured by the JBI critical appraisal tool). Further, there was heterogeneity in terms of secondary prevention components, content delivery, intensity and outcomes measured between and within the three different modes of e-Health delivery.

In summary, web-based technology applicable for desktop or laptop computers was the most frequently used mode of e-Health delivery. Eight studies delivered m-Health secondary prevention programmes. Unlike that described in previous reviews [10, 11], seven studies combined web-based and m-Health technology. This finding shows a trend towards utilising all available technology when designing and implementing e-Health secondary prevention programmes for patients with CAD [10, 52]. Further, the acceptability of the combination of m-Health and web-based technology was high compared whit web-based technology alone, which varied substantially. However, the majority of the studies combining m-Health and web-based technology, delivered the programme as an adjunct to traditional CR and/or usual care [29, 31, 33–35, 40], which could have affected the acceptability in relation to the in-person approaches. Of those studies, all reported some level of effectiveness in at least one of three outcome categories defined a priori. A previous meta-analysis [12] reported that telehealth delivered in combination with traditional CR showed favourable changes in secondary prevention for patients with CAD of medium and long-term duration. However, many of those studies were telephone-based and the numbers of participants in traditional CR was moderate to low [12]. Notably, another previous review that assessed evidence from an e-Health secondary prevention programme versus traditional centre-based CR for CAD, found no difference in outcomes between the two modes of delivery [8]. These reviews recommended that e-Health could be offered to patients who cannot attend traditional CR [8] or as an adjunct [12]. It has also been described that e-Health secondary prevention programmes would decrease non-participation and the dropout rate due to better adaptation to the patients’ needs and preferences [7]. Therefore, based on knowledge of barriers associated with non-participation and dropout from traditional secondary prevention programmes, more research is needed to develop feasible modes of e-Health delivery which provide increased flexibility in relation to time and geographical location.

In terms of secondary prevention programme components, this review demonstrated that health education was the most frequently provided secondary prevention component. This differs from older reviews, which report that physical activity and exercise management were most frequently evaluated [8, 16]. This development is in line with the updated document from BACPR, which recognises that the educational component remains fundamental to all other secondary prevention programme components to increase self-management and healthy behaviour [15, 19]. Utilisation of e-Health technology in education delivery provides easier access and patients can self-pace through educational materials. However, patient-related barriers such as low electronic health literacy are of importance to the patients’ ability to apply knowledge, make appropriate decisions and achieve better self-management behaviour [2]. None of the studies that included health education assessed in this review aimed to specifically address the electronic health literacy skills needed in an e-Health context, although, one web-based programme [41] reported positive improvement in self-efficacy, which is an indicator of health literacy [53]. However, an m-Health secondary prevention programme based on self-efficacy theory reported non-significant improvement in medication self-efficacy. The intervention delivered consisted of medication reminders and health education through text messages [37]. The lack of improvement may be associated with psychosocial factors and low socioeconomic status, which are reported as barriers for digital health adoption [2].

This review identified two web-based studies and four studies combining web-based and m-Health that report positive effects on psychosocial outcomes. It may be discussed whether psychosocial health management gets too little attention in the development of e-Health secondary prevention programmes. Despite this, it is well known that depression, anxiety and low quality of life are common among patients with CAD [4, 26] and psychosocial health is recommended as a standard component in secondary prevention programmes [4, 15]. Psychosocial factors cannot be underestimated in terms of their impact on medication adherence, behavioural change and self-management. One study that aimed to reduce negative emotions by providing advice about stress and anxiety management skills and social support [41] reported favourable changes in emotional and social quality of life. The study demonstrates that a web-based mode of psychosocial management and support delivery can be feasible in secondary prevention for CAD. Further, this review has identified viable tools, such as feedback messages, chat sessions and online discussion groups, that employ web-based technology to replace face-to-face meetings. To improve health outcomes, the e-Health system should be designed to foster effective interactions between patients and health professionals. However, little is known about whether social support offered through e-Health programmes has the same effect on self-management behaviour and psychosocial outcomes as traditional secondary prevention programmes [54].

In terms of medication adherence, a previous review identified seven positive RCTs published in the period 2012–2015 [55]. This review identified an additional four studies that utilis m-Health interventions published after 2015 [27, 28, 30, 32], of which one study reported a positive trend for medication adherence [30]. This finding shows a potential for using m-Health to improve medication adherence. According to studies providing a combination of m-Health and web-based technology, one study reported a positive short-term effect on medication adherence [33]. It is surprising however, that studies combining m-Health and web-based technology did not utilise the technology available by providing medical risk management. This finding shows a need for more rigorous studies utilising the combination of different modes of e-Health technology available to improve medication adherence.

Within the mode of m-Health delivery positive effects were reported on physical activity [27, 28] and smoking cessation [28] in the TEXT ME trial. To our knowledge, no previous reviews evaluating the effect of m-Health secondary prevention programmes on cardiovascular diseases have reported studies demonstrating positive effects on smoking cessation [55, 56]. Unlike other m-Health interventions assessed, the TEXT ME trial was based on the behavioural change technique, which may be a determining factor for success. Interventions based on behavioural change theory tend to be more effective than those without a theoretical framework [57]. Using principles from behaviour change theories in the development of m-Health secondary prevention programmes may increase the likelihood of success [58]. A number of theoretical frameworks offer guidance on how to manage mechanisms of change in individual health-related behaviour patterns in the development of complex interventions. However, it is unclear whether and how the use of theory influences the effectiveness of e-Health interventions [57], and most studies assessed in this review did not clearly describe the underlying theoretical framework. Therefore, more studies that apply a theoretical framework that is made explicit in the reporting are needed. Several useful frameworks exist. For example, the UK Medical Research Council’s (MRC) revised framework [59] can be used to guide the complex e-Health secondary programme development. Another alternative could be the eHealth Enhanced Chronic Care Model (Model (eCCM). The eCCM is a further development of the Chronic Care Model (CCM) that included self-management support and clinical information systems as two of its key elements in order to provide technological skills, access to data, information and knowledge needed to improve health [54]. Further, since it is proposed that behaviour change occurs when patients are well informed, highly motivated and have the skills necessary to perform self-management behaviour [60], e-Health literacy is of relevance. The e-Health literacy model aims to empower patients and enable them to fully participate in health decisions informed by different modes of e-Health delivery [53], and could be an alternative when developing health education as a component of e-Health secondary prevention programmes.

The strengths of this review include its systematic approach to data collection. The Cochrane Consumers and Communication Review Group’s data extraction template [22] was used to capture all relevant information about the studies included. Study characteristics were extracted using the TIDieR checklist [23]. The quality appraisal was systematically assessed by two independent researchers (GB and TRP) using the JBI critical appraisal tool for RCTs [24]. PRISMA guidelines [17] for reporting systematic reviews were used to minimise potential sources of bias. Articles identified through referenced and hand searches were also considered for inclusion. Nevertheless, the review was limited to empirical research published in the English language. There may also be unpublished theses or conference proceedings that were overlooked. Furthermore, as the literature search did not yield any results from Latin America or Sub-Saharan Africa, no studies from these countries were included in this review, thus reducing the generalisability of the results for these populations. However, this systematic review is merely the first step towards developing new and innovative e-Health interventions relating to follow-up care for patients with CAD.

## Conclusion

This systematic review shows that evidence exists to support the use of e-Health secondary prevention programmes for patients with CAD. However, comparison across studies highlighted a wide variability of secondary prevention programme components and outcomes within the different modes of delivery. High quality trials are needed to define the most efficient mode of delivery and components capable of addressing a favourable outcome for patients.

## Additional files

Additional file 1: **Table S1.** Search strategy carried out in Medline and adapted to carry out similar searches in CINAHL and Embase. Search date: 15.03.2018. (DOCX 13 kb)

## Abbreviations

6MWT: Six minute walk test
AOR: Adjusted Odds Ratio
BACPR: British Association of Cardiovascular Prevention and Rehabilitation
BMI: Body mass index
CAD: Coronary artery disease
CINAHL: Cumulative Index to Nursing and Allied Health
CR: Cardiac rehabilitation
EACPR: European Association for Cardiovascular Prevention and Rehabilitation
eCCM: eHealth Enhanced Chronic Care Model
e-Health: electronic health
EQ5D: Euroqol 5 Dimensions Index
ESC: European Society of Cardiology
GPAQ: Global Physical Activity Questionnaire
GSES: General Self-Efficacy Scale
iCBT: internet-Based Cognitive Therapy
JBI: Joanna Briggs Institute
K10: Kessler 10 (K10) Psychological Distress Scale
MARS-5: Medication Adherence Report Scale
m-Health: mobile health
MMAS-8: Morisky Medication Adherence Scale; MRC: UK Medical Research Council; MTT Maximal time on the treadmill stress test
PRISMA: Preferred Reporting Items for Systematic Reviews and Meta-Analyses
PROSPERO: International Prospective Register of Systematic Reviews
PVO2: Peak oxygen uptake
RCTs: Randomized controlled trials
SF-36: Short Form Health Survey 36
TIDieR: Template for Intervention Description and Replication
