# Supplementary material for: Modes of e-Health delivery in secondary prevention programmes for patients with coronary artery disease: a systematic review
Source: BMC Health Serv Res. 2019 Jun 10;19:364. doi: 10.1186/s12913-019-4106-1 (PMC6558849; doi:10.1186/s12913-019-4106-1)
Supplement: Supplementary file 1 — Table S1. Search strategy carried out in Medline and adapted to carry out similar searches in CINAHL and Embase. Search date: 15.03.2018. (DOCX 13 kb) [file 12913_2019_4106_MOESM1_ESM.docx]

**Supplementary Table 1:** Search strategy carried out in Medline and adapted to carry out similar searches in CINAHL and Embase. Search date: 15.03.2018.

| 1 | exp Myocardial ischemia |
| --- | --- |
| 2 | exp Ischemic heart disease |
| 3 | Myocardial ischemia.tw |
| 4 | Angina pectoris.tw |
| 5 | Myocardial infarction.tw |
| 6 | Coronary disease.tw |
| 7 | (1 OR… OR 6) |
| 8 | exp Computers & computerization |
| 9 | exp Software |
| 10 | exp Telecommunication |
| 11 | exp Computers |
| 12 | exp Virtual reality |
| 13 | exp Internet |
| 14 | exp Telenursing |
| 15 | Ehealth.tw OR E-health.tw |
| 16 | Mhealth.tw OR M-health.tw |
| 17 | Chatbot*.tw |
| 18 | Software.tw |
| 19 | Telecommunication*.tw |
| 20 | Computer*.tw |
| 21 | Virtual realit*.tw |
| 22 | Internet.tw |
| 23 | Telenurs*.tw |
| 24 | Mobile app*.tw |
| 25 | Video gam*.tw |
| 26 | Smartphone*.tw |
| 27 | User computer interface*.tw |
| 28 | Telemed*.tw |
| 29 | Text messag*.tw |
| 30 | Telerehab*.tw |
| 31 | Telehealth.tw |
| 32 | Artificial voice*.tw |
| 33 | Text to speech.tw |
| 34 | (8 OR … OR 33) |
| 35 | exp Patient care |
| 36 | exp Rehabilitation |
| 37 | exp Secondary prevention |
| 38 | exp Self care |
| 39 | exp Health education |
| 40 | Rehab*.tw |
| 41 | Secondary prevent*.tw |
| 42 | Self management.tw |
| 43 | Nurs* intervention*.tw |
| 44 | Health education.tw |
| 45 | (35 OR… OR 44) |
| **Combined search** |  |
| 45 | (7 AND 34 AND 45) |
| **Applied filters** | (2003-now); English |
